# Toward elimination of mother–to–child transmission of HIV in South Africa: how best to monitor early infant infections within the Prevention of Mother–to–Child Transmission Program

**DOI:** 10.7189/jogh.07.010701

**Published:** 2017-06

**Authors:** Gayle G Sherman, Ahmad Haeri Mazanderani, Peter Barron, Sanjana Bhardwaj, Ronelle Niit, Margaret Okobi, Adrian Puren, Debra J Jackson, Ameena Ebrahim Goga

**Affiliations:** 1 Centre for HIV and STI, National Institute for Communicable Diseases, a division of the National Health Laboratory Services, Johannesburg, South Africa; 2 Department of Paediatrics and Child Health, Faculty of Health Sciences, University of the Witwatersrand, Johannesburg, South Africa; 3 Department of Medical Virology, University of Pretoria, Pretoria, South Africa; 4 School of Public Health, Faculty of Health Sciences, University of the Witwatersrand, Johannesburg, South Africa; 5 United Nations Children’s Fund, Pretoria, South Africa; 6 Nelson Mandela University, Port Elizabeth; and University of South Africa, Pretoria, South Africa; 7 Department of Biology, Massachusetts Institute of Technology, Cambridge, Massachusetts, USA; 8 Division of Virology and Communicable Diseases, School of Pathology, University of the Witwatersrand Medical School, Johannesburg, South Africa; 9 School of Public Health, University of the Western Cape, Cape Town, South Africa; 10 United Nations Children’s Fund, New York, New York, USA; 11 Health Systems Research Unit, South African Medical Research Council, Cape Town, South Africa; 12 Department of Paediatrics, University of Pretoria, Pretoria, South Africa

## Abstract

**Background:**

South Africa has utilized three independent data sources to measure the impact of its program for the prevention of mother–to–child transmission (PMTCT) of HIV. These include the South African National Health Laboratory Service (NHLS), the District Health Information System (DHIS), and South African PMTCT Evaluation (SAPMTCTE) surveys. We compare the results of each, outlining advantages and limitations, and make recommendations for monitoring transmission rates as South Africa works toward achieving elimination of mother–to–child transmission (eMTCT).

**Methods:**

HIV polymerase chain reaction (PCR) test data, collected between 1 January 2010 to 31 December 2014, from the NHLS, DHIS and SAPMTCTE surveys were used to compare early mother–to–child transmission (MTCT) rates in South Africa. Data from the NHLS and DHIS were also used to compare early infant diagnosis (EID) coverage.

**Results:**

The age–adjusted NHLS early MTCT rates of 4.1% in 2010, 2.6% in 2011 and 2.3% in 2012 consistently fall within the 95% confidence interval as measured by three SAPMTCTE surveys in corresponding time periods. Although DHIS data over–estimated MTCT rates in 2010, the MTCT rate declines thereafter to converge with age–adjusted NHLS MTCT rates by 2012. National EID coverage from NHLS data increases from around 52% in 2010 to 87% in 2014. DHIS data over–estimates EID coverage, but this can be corrected by employing an alternative estimate of the HIV–exposed infant population.

**Conclusion:**

NHLS and DHIS, two routine data sources, provide very similar early MTCT rate estimates that fall within the SAPMTCTE survey confidence intervals for 2012. This analysis validates the usefulness of routine data sources to track eMTCT in South Africa.

Since 2004, when South Africa officially launched its program to prevent mother–to–child transmission (PMTCT) of HIV, huge strides have been made in curbing the incidence of infant HIV infection. Despite the national antenatal HIV sero–prevalence, as measured among women attending public health facilities, consistently remaining between 29% and 31% since 2004, the number of vertically infected infants has continued to decrease [1–3]. Various methods utilizing different data sets have been employed to monitor the effectiveness of the PMTCT program. Data from the National Health Laboratory Service (NHLS) and the District Health Information System (DHIS) have been used independently to monitor the early mother–to–child transmission (MTCT) rate and the coverage of early infant diagnosis (EID) testing among the HIV–exposed infant population. Furthermore, three national, facility–based South African PMTCT Evaluation (SAPMTCTE) surveys have been conducted since 2010 to assess the effectiveness of the national PMTCT program. We compare the results of each method, outlining their respective advantages and limitations, and make recommendations as South Africa prepares for pre–validation of its elimination of mother–to–child transmission (eMTCT) status [4].

## Elimination of mother–to–child transmission

Criteria and processes for validation of eMTCT of HIV have been suggested by the World Health Organization (WHO) [4]. These minimum global standards refer to specific impact and process targets which need to be met prior to certification of eMTCT. The required impact targets are ≤50 new pediatric HIV infections per 100 000 live births and a transmission rate of either <5% in breastfeeding populations or <2% in non–breastfeeding populations.^4^ Countries are encouraged to apply for validation of eMTCT once impact targets have successfully been met for one year, process targets for two years, and eMTCT has been achieved in at least one of the lowest–performing sub–national administrative units. In order to achieve eMTCT targets, appropriate monitoring tools need to inform a national validation report that must subsequently be approved by national, regional and global validation committees.

## South African Guidelines for Early Infant Diagnosis

In 2004, the South African National Department of Health (NDOH) recommended routine HIV PCR testing in HIV–exposed infants at six weeks of age [5]. Since then, a number of important changes have been made to EID guidelines. Whereas testing symptomatic infants prior to six weeks of age and testing all HIV–exposed infants at six weeks of age has been standard of care for over a decade, in 2013 the additional targeted birth testing of asymptomatic but “high–risk” infants was implemented in some parts of the country [6]. Subsequently, in June 2015, as a means of ensuring earlier detection of intra–uterine infected infants, routine birth testing for all HIV–exposed infants was introduced into national guidelines [5]. Additional changes to EID guidelines include a second HIV PCR test at 10 weeks of age for those who test negative at birth and the falling–away of the standard six–week test (**Table 1**). Furthermore, the testing method for confirming HIV infection status has changed from an HIV viral load to a confirmatory HIV PCR test [5,8]. These changes in EID guidelines have important implications for monitoring MTCT using routine laboratory data.

**Table 1:** South African National Guidelines for Early Infant Diagnosis of HIV Exposed Infants [3,5–10]

Year of Guideline	2004	2008	2010	2013	2015
**If HIV–exposed & symptomatic:**	HIV PCR test at presentation				
**If HIV–exposed & asymptomatic:**	HIV PCR at ≥6 wks	HIV PCR at 6 wks	HIV PCR at 6 wks	HIV PCR at 6 wks	HIV PCR at birth
**If HIV PCR positive:**	HIV VL test at baseline Repeat HIV PCR only if child is asymptomatic	HIV VL test at baseline Repeat HIV PCR only if child is asymptomatic	Confirmatory HIV VL: VL >10 000 cps/ml confirms HIV positive status	Confirmatory HIV VL: Any quantified VL confirms HIV positive status	Confirmatory HIV PCR
**If HIV PCR negative:**	Repeat HIV PCR test if infant symptomatic, and repeat 6 wks after cessation of breastfeeding				
					Repeat HIV PCR at 10 wks
					Repeat HIV PCR at 18 wks (if received 12 wks NVP)
					Repeat HIV PCR if breastfeeding and maternal VL >1000 cps/ml

PCR – polymerase chain reaction, cps/ml – copies per milliliter, wks – weeks, VL – viral load, NVP – Nevirapine

## Data sources used to monitor MTCT in the PMTCT program

Early MTCT refers to vertical transmission that is acquired either intra–uterine or intrapartum, and is typically monitored between the ages of four to eight weeks of age. In South Africa, early MTCT has been measured using different methodologies from three different data sources, namely the NHLS, the DHIS and three SAPMTCTE surveys. In addition, uptake of early infant testing has been calculated from both NHLS and DHIS data.^1^

The NHLS provides diagnostic services for the whole of the public health sector in South Africa, estimated at 80% of the total population of the country. Every laboratory test is accompanied by an NHLS test requisition form that stipulates identifying details for each patient, the date of specimen collection, the facility at which testing was performed and the type of test requested. This information is entered into the laboratory information system (LIS) together with the test results and stored centrally in the NHLS corporate data warehouse (CDW). Monthly reports are generated detailing the number of HIV PCR tests performed and the number of HIV PCR positive test results for approximately 4000 health care facilities across the country.

The DHIS gathers aggregate data from all health care facilities in each of the 52 health districts in South Africa, and includes HIV PCR results from the NHLS. These data are collected and summated in prescribed registers at each facility. They are then captured electronically in the DHIS and transmitted to provincial and national level for collation. There is monthly reporting at sub–district, district, provincial and national level to track health service delivery. The data elements collected include those that make up the PMTCT indicators.

The SAPMTCTE were national surveys conducted over three consecutive years by the South African Medical Research Council, with the aim of determining the impact of the PMTCT program using a population–based representative sample. The sampling unit of these surveys was primary level clinics reporting at least 130 first DTP immunisations per year [11]. The primary objective of the SAPMTCTE surveys was to determine MTCT of HIV at 6 weeks of age and more recently at 3, 6, 9, 12, 15 and 18 months postpartum. Three surveys have been conducted to date from June to December 2010, August 2011 to March 2012 and October 2012 to May 2013.

## METHODS

HIV PCR test data collected between 1 January 2010 and 31 December 2014 from three different sources, the NHLS, the DHIS and SAPMTCTE surveys, were used to compare early MTCT rates across five years (2010–2014). For the SAPMTCTE surveys, the MTCT rates and their 95% confidence intervals were allocated to the year in which each survey was initiated. In addition to early MTCT rates, data from the NHLS and DHIS were used to compare EID coverage.

### Early MTCT rates

The NHLS CDW reports the HIV PCR positivity rates in children <2 months of age as a proxy for early MTCT rates by calculating the proportion of HIV PCR positive tests to the total number of HIV PCR tests performed in this age group.

The DHIS indicator used to monitor positivity in HIV–exposed infants around 6 weeks is the “infant first PCR test positive around 6 weeks rate” with a numerator of “infant first PCR positive around 6 weeks” and a denominator of “infant first PCR test conducted around 6 weeks”. Around 6 weeks is defined as an infant that is first tested between the ages of 4 and 12 weeks.

The SAPMTCTE surveys enrolled infants attending their 6–week immunisation visit, if they were between 4 and 8 weeks of age, regardless of their mother’s HIV infection status and collected dried blood spot (DBS) samples from them. HIV–exposed infants and HIV–infected infants were defined as those that tested DBS HIV ELISA and DBS HIV PCR positive, respectively. The early MTCT was calculated using the number of HIV PCR positive infants as the numerator and DBS HIV ELISA positive with HIV PCR result, as a denominator.

Because the ages of the infants used to calculate early MTCT in the three data sets differed, NHLS CDW data was re–extracted to match the age of HIV PCR testing in the DHIS data, 4–12 weeks of age, and in the SAPMTCTE surveys, 4–8 weeks of age.

### Early Infant Diagnosis coverage

The NHLS CDW defines EID coverage as the number of registered HIV PCR tests in infants aged ≤2 months of age divided by the expected number of HIV–exposed infants, expressed as a percentage [1]. The denominator (ie, the HIV–exposed population requiring HIV testing) is calculated using the national registered live births published by STATS SA multiplied by the national maternal antenatal sero–prevalence of HIV reported by the NDOH [3,12].

The DHIS indicator used to monitor EID coverage is the “infant first PCR test around 6 weeks uptake rate” with a numerator of “infant first PCR test conducted around 6 weeks” and a denominator of “live births to HIV positive women”. To account for potential under–reporting of infants born to HIV–infected mothers in DHIS, an alternative denominator is also used that estimates HIV–exposed infant population by multiplying ‘total live births to all women’, captured by DHIS, with national maternal antenatal HIV sero–prevalence, reported by the NDOH [3].

## RESULTS

### Early MTCT rates

The early MTCT rates as determined from NHLS data compare closely with results from the SAPMTCTE surveys, differing by 0.7% in 2010, 0.0% in 2011, and –0,2% in 2012 (**Table 2**). When the NHLS data are re–adjusted to only include HIV PCR tests performed for the same age ranges as the SAPMTCTE surveys (ie, infants 4–8 weeks of age), the NHLS early MTCT rate per year from 2010–2014 decreases uniformly by 0.1% (**Table 2**, Data set 4). Importantly, after matching the age ranges of infants in the NHLS and SAPMTCTE data, the NHLS early MTCT rates consistently fall within the 95% confidence interval as measured by three SAPMTCTE surveys in corresponding time periods (**Table 2**, Data set 3).

**Table 2:** Early HIV transmission rates in South Africa 2010–2014*

	Data sets	Age	2010	2011	2012	2013	2014
1)	NHLS HIV PCR tests	<2months	119 808	164 181	184 400	195 188	222 559
	NHLS HIV PCR+ tests	<2months	5282	4609	4440	3912	4054
	NHLS % positive HIV PCR tests	<2months	4.2%	2.7%	2.4%	2.0%	1.8%
2)	DHIS HIV PCR tests	±6 weeks	178 241	211 942	237 869	243 786	247 037
	DHIS HIV PCR+ tests	±6 weeks	17 528	9556	6611	5184	4089
	DHIS % positive HIV PCR tests	±6 weeks	9.0%	4.3%	2.7%	2.1%	1.6%
3)	SAPMTCTE MTCT rate (95% confidence intervals)	4–8 weeks	3.5% (2.9%–4.1%)	2.7% (2.1–3.2%)	2.6% (2.0–3.2%)		
4)	NHLS HIV PCR tests	4–8 weeks	113 722	157 411	176 787	186 969	208 364
	NHLS HIV PCR+ tests	4–8 weeks	4849	4271	4110	3579	3624
	NHLS % positive HIV PCR tests	4–8 weeks	4.1%	2.6%	2.3%	1.9%	1.7%
5)	NHLS HIV PCR tests	4–12 weeks	139 517	187 020	206 990	216 410	236 708
	NHLS HIV PCR+ tests	4–12 weeks	7158	6125	5823	5064	5106
	NHLS % positive HIV PCR tests	4–12 weeks	4.9%	3.2%	2.7%	2.3%	2.1%

NHLS –National Health Laboratory Service, PCR – polymerase chain reaction, SAPMTCTE – South African PMTCT Evaluation, , DHIS – District Health Information System

*Comparison of MTCT rates for NHLS, DHIS and SAPMTCTE are tabulated in data sets 1)–3). In addition to the routine NHLS data reported at 1) <2months of age, NHLS data are presented at ages 4) 4–8 weeks and 5) 4–12 weeks, to more closely approximate the eligible age group in the SAPMTCTE surveys and reported age group in the DHIS data respectively.

The number of HIV PCR tests as recorded by the DHIS is consistently higher than for NHLS data (**Table 2**). The same is true for the calculated early MTCT rate, except for the 2014 estimate. The DHIS early MTCT rate was reported as more than double the NHLS and SAPMTCTE MTCT rates in 2010 (**Table 2**). However, this rate falls dramatically over the next two years to lie within the SAPMTCTE survey’s 95% confidence interval for 2012.

When the NHLS data was adjusted to include HIV PCR tests performed on infants 4–12 weeks of age, the early MTCT rates for all years increased (**Table 2**, Data set 5). In 2013 and 2014 the NHLS early MTCT rates were higher than the DHIS rates by 0.2% and 0.5%, respectively (**Table 2**).

### Early Infant Diagnosis coverage

**Figure 1:**
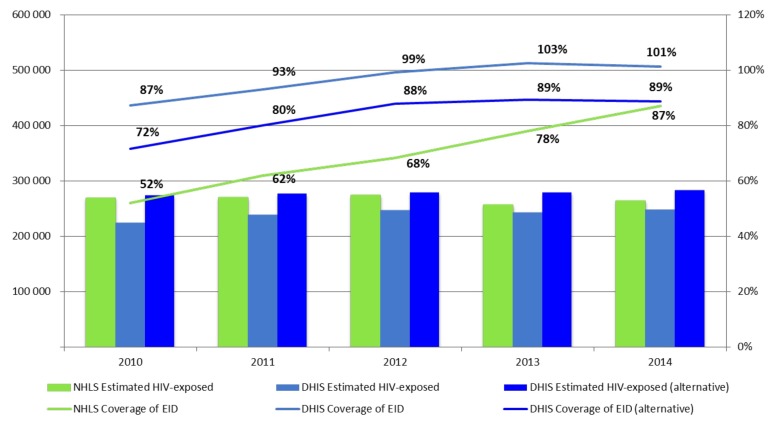
Early Infant Diagnosis Coverage rates in South Africa 2010–2014. The bar graph represents the population of HIV-exposed infants as estimated by NHLS, DHIS and from an alternative DHIS calculation and the line graphs represent the coverage of EID for each data set.

National EID coverage as determined from NHLS data are seen to increase steadily from 52% in 2010 to 68% in 2012 and 87% in 2014 (**Figure 1**). Alternatively, EID coverage as reported from DHIS data appears to be consistently higher and exceeds 100% by 2013. However, when the alternative DHIS denominator is used, EID coverage converges with that reported by the NHLS over time with only a 2% difference in 2014 (**Figure 1**).

Early MTCT and EID coverage rates between 2010 and 2014 showed similar patterns to the national one for all nine provinces (data not shown).

## DISCUSSION

This analysis shows that data from routine sources, namely the NHLS and DHIS, produce similar results for monitoring the effectiveness of South Africa’s PMTCT program. The accuracy of the early MTCT rates calculated from these distinct routine data sets was verified by comparing them to the SAPMTCTE surveys conducted in the same year, demonstrating that results from routine data sources lie within the 95% confidence intervals of the surveys.

The differences between DHIS and NHLS data in 2010–2012, and the remarkable reduction in MTCT in DHIS data are likely attributable to quality improvement training in the field after it was found that health care workers were reporting the DHIS indicators incorrectly (ie, reporting all HIV PCR tests performed on children of all ages instead of reporting the first HIV PCR test performed on infants aged around 6 weeks) [13].

By 2012, the higher number of HIV PCR tests recorded by the DHIS likely reflects an inclusion of 8–12 week old infants as compared with the NHLS data that excluded this age group and included the 0–4 week age group where very little HIV PCR testing was performed. From 2013, when targeted birth testing of neonates was introduced into the South African guidelines, the decreasing difference in the number of HIV PCR tests performed according to DHIS and NHLS is likely attributable to neonatal testing being included in the NHLS data but excluded from the DHIS data because no mechanism was yet in place for health care workers to report on birth testing. Furthermore, it is postulated that an increase in confirmatory HIV PCR testing, resulting in double counting, may be the explanation for the higher early MTCT rates in 2013 and 2014 in the NHLS vs the DHIS data.

Both NHLS and DHIS document an increase in infant HIV testing coverage between 2010 and 2014 with a convergence in both the absolute numbers tested as well the overall coverage rates.

The use of multiple methodologies to monitor the same PMTCT targets was initially needed to measure national PMTCT effectiveness. The similarities between survey results and routine data sources obviates the urgent need for continued, regular parallel surveillance activities that are expensive and resource and labor intensive.

### Limitations

There are clear requirements and limitations to using routine NHLS and DHIS data to monitor early MTCT rates and EID coverage, as well as limitations to the SAMTCTE surveys to calculate MTCT rates.

The use of routine laboratory data to monitor the PMTCT program requires accurate information to be provided on laboratory requisition forms and reliable data capturing from the requisition forms into the LIS. Importantly, for this data to be a true reflection of the country’s MTCT rate there must be close to 100% testing coverage, close to zero missed diagnostic opportunities in the laboratory and accurate collection of age data [14]. Missed diagnostic opportunities are defined as samples yielding neither a positive or negative result related to pre–analytical (eg, insufficient sample for processing) and analytical errors (eg, indeterminate or invalid results). A further important limitation is that there is currently no unique identifier for individual patients and no accurate means of de–duplicating test result data. Therefore, infants with multiple HIV PCR tests cannot be distinguished from infants with a single HIV PCR test. Whereas in the past it was assumed that very few infants would access more than one HIV PCR test by 2 months of age, current guidelines recommend confirmatory HIV PCR testing for those infants who test positive and repeat testing for those infants who are symptomatic. Hence, reliable MTCT rates, including post–natal transmission, can no longer be calculated from routine NHLS data without the introduction of unique patient identifiers. The NDOH has communicated that unique patient identifiers will likely be implemented in all public sector facilities in the 2016/17 financial year (M Wolmarans, Chief Director, Strategic Planning, NDOH. Personal communication, February 15, 2016).

Regarding calculating EID coverage from NHLS data, in addition to challenges in de–duplicating data as noted above, limitations include calculating the number of HIV exposed infants requiring testing (ie, the denominator) from national antenatal maternal sero–prevalence data and STATS SA registered live birth data. As these are published after a lag of 2–3 years, EID coverage for 2014 has been calculated based on the ANC Maternal Sero–prevalence data from 2013 and, hence, may not be accurate. Since a proportion of live births registered are from the private health care sector, the number of HIV–exposed infants may be overestimated accounting for a lower EID coverage.

Limitations to the use of DHIS data primarily relate to training health care workers to capture the correct information in a consistent manner. While this undoubtedly has improved over recent years, with practical data improvement interventions found to significantly increase the completeness and accuracy of the data used to monitor PMTCT services in South Africa, there remain a great number of challenges [15]. For instance, because an unknown number of women deliver without any or recent HIV testing or do not disclose their HIV positive status to health care workers in the labor ward, the “live births to HIV positive women” is likely under reported. Hence, the denominator used to calculate EID coverage is too low resulting in an over–estimation of coverage that exceeds 100% by 2013 [16]. As demonstrated, this can be addressed by using an alternative denominator to estimate the HIV–exposed infant population. Data from the SAPMTCTE survey further support the likelihood that determining the HIV–exposed infant population from maternal history taking will over–estimate EID coverage. The SAPMTCTE survey’s found that 3–4% of HIV positive women did not report being positive, either because they did not know their status, for reasons which include seroconversion during pregnancy, or chose not to disclose [11].

Limitations of the SAPMTCTE survey primarily relate to sampling. The surveys provide data for healthy infants presenting for immunisation only, excluding infants who were ill at the first immunisation visit, those who had failed to present for immunisation, and those who had died by 6 weeks of age. Hence, the point–estimate is likely an under–estimation of true early infant HIV infection prevalence [17].

### Advantages and disadvantages

Whereas each methodology has its own advantages, disadvantages and challenges, it is important to appreciate that there are certain differences between the data sources that cannot be controlled for. Both DHIS and NHLS record HIV PCR data for all infants known to be HIV exposed and tested whereas the SAPMTCTE surveys exclude certain groups of infants who are possibly at high risk of HIV–infection [18]. On the other hand, the SAPMTCTE surveys tested all infants for HIV–exposure and are therefore inclusive of mothers who do not report being HIV positive. Whereas repeat HIV PCR tests on the same patient are included in both the DHIS and NHLS data, the SAPMTCTE surveys do not include duplicate testing.

Clear advantages of using NHLS data are that it allows for near real–time monitoring of early MTCT and EID coverage and comes at very little additional cost. In contrast, DHIS data takes time to collate and comes at much greater expense. Conversely, a distinct disadvantage of the NHLS CDW to DHIS is that the LIS does not hold clinical data. While the SAPMTCTE surveys are likely to provide the most accurate data, they are expensive and time consuming. Ideally, national–level surveys should be conducted periodically; reserved for validating routine data sources; and answering specific, more detailed questions where no other data are available. Examples include determining the rate of linking HIV PCR positive infant to care, and the interval between infant diagnosis and initiation of treatment.

### The way forward

There is undoubtedly scope for improvement in the accurate and timely reporting of PMTCT targets required to achieve elimination. By merging DHIS and NHLS CDW data, a streamlined and efficient method could be forged from current routine monitoring activities. Although the LIS does not hold clinical data, there are opportunities of addressing this by incorporating NHLS requisition forms with the appropriate clinical data populated within the CDW. This would enable clinical data from across the PMTCT cascade to be captured, including data on maternal treatment regimens, infant linkage into care, infant treatment initiation and retention in care. This will preclude the unnecessary duplication of data capturing among health care workers of laboratory data and provide a more robust data set from which to monitor the effectiveness of the PMTCT program at all levels of health care delivery. A consolidated monitoring tool will, nevertheless, pose certain challenges. In particular, the accurate and consistent capturing of a prescribed minimum clinical data set will need to be strictly adhered to.

Access to accurate clinical information will be important for documenting the process targets for eMTCT validation particularly as South Africa recommends breastfeeding but has no system for monitoring final MTCT rates. Furthermore, a unique patient identifier, employed from birth, (eg, printing patient–retained immunisation booklets with unique barcodes that can be captured within the LIS) will be a prerequisite in order to accurately calculate MTCT rates using routine laboratory data. These challenges will be easier to overcome if there is a consolidated effort between clinical and laboratory personnel and a clear directive from the NDOH.

The effectiveness of South Africa’s PMTCT program, as determined by the early MTCT rate and EID coverage, has been monitored in parallel using routine laboratory data and operational data collected by each district in the country. Additionally, three national surveys have been conducted between 2010 and 2012, evaluating the effectiveness of the PMTCT program on early MTCT. All three methodologies provide very similar early MTCT rates, with the laboratory and DHIS estimates falling within the survey confidence intervals. These surveys validate the accuracy, and therefore usefulness, of routine data sources, and raise questions about the continued value of regular parallel surveillance activities. As recent changes in national EID guidelines pose new challenges to the accuracy of both NHLS and DHIS data, the continued value of SAPMTCTE surveys will be in periodically validating routine data methods. By introducing unique patient identifiers and consolidating clinical information within the LIS, a more efficient method of monitoring the effectiveness of the national PMTCT program using routine laboratory data are envisaged. This will not only preclude unnecessary duplication of data capturing within the DHIS but also reliably inform eMTCT targets. By outlining the value of routine laboratory data, it is anticipated that these findings will inform South Africa’s pediatric HIV surveillance systems as well as other countries monitoring early MTCT rates and EID coverage.
